# Gait analysis system for assessing abnormal patterns in individuals with hemiparetic stroke during robot-assisted gait training: a criterion-related validity study in healthy adults

**DOI:** 10.3389/fnbot.2025.1558009

**Published:** 2025-05-21

**Authors:** Issei Nakashima, Daisuke Imoto, Satoshi Hirano, Hitoshi Konosu, Yohei Otaka

**Affiliations:** ^1^Department of Rehabilitation Medicine, School of Medicine, Fujita Health University, Aichi, Japan; ^2^Toyota Motor Corporation, Aichi, Japan; ^3^Department of Rehabilitation, Fujita Health University Hospital, Aichi, Japan; ^4^Department of Rehabilitation Medicine, Graduate School of Medicine, Fujita Health University, Aichi, Japan

**Keywords:** abnormal gait, cerebrovascular disease, gait analysis, hemiparetic stroke, rehabilitation

## Abstract

**Introduction:**

Gait robots have the potential to analyze gait characteristics during gait training using mounted sensors in addition to robotic assistance of the individual’s movements. However, no systems have been proposed to analyze gait performance during robot-assisted gait training. Our newly developed gait robot,” Welwalk WW-2000 (WW-2000)” is equipped with a gait analysis system to analyze abnormal gait patterns during robot-assisted gait training. We previously investigated the validity of the index values for the nine abnormal gait patterns. Here, we proposed new index values for four abnormal gait patterns, which are anterior trunk tilt, excessive trunk shifts over the affected side, excessive knee joint flexion, and swing difficulty; we investigated the criterion validity of the WW-2000 gait analysis system in healthy adults for these new index values.

**Methods:**

Twelve healthy participants simulated four abnormal gait patterns manifested in individuals with hemiparetic stroke while wearing the robot. Each participant was instructed to perform 16 gait trials, with four grades of severity for each of the four abnormal gait patterns. Twenty strides were recorded for each gait trial using a gait analysis system in the WW-2000 and video cameras. Abnormal gait patterns were assessed using the two parameters: the index values calculated for each stride from the WW-2000 gait analysis system, and assessor’s severity scores for each stride. The correlation of the index values between the two methods was evaluated using the Spearman rank correlation coefficient for each gait pattern in each participant.

**Results:**

The median (minimum to maximum) values of Spearman rank correlation coefficient among the 12 participants between the index value calculated using the WW-2000 gait analysis system and the assessor’s severity scores for anterior trunk tilt, excessive trunk shifts over the affected side, excessive knee joint flexion, and swing difficulty were 0.892 (0.749–0.969), 0.859 (0.439–0.923), 0.920 (0.738–0.969), and 0.681 (0.391–0.889), respectively.

**Discussion:**

The WW-2000 gait analysis system captured four new abnormal gait patterns observed in individuals with hemiparetic stroke with high validity, in addition to nine previously validated abnormal gait patterns. Assessing abnormal gait patterns is important as improving them contributes to stroke rehabilitation.

**Clinical trial registration:**

https://jrct.niph.go.jp, identifier jRCT 042190109.

## 1 Introduction

Improving gait of individuals with hemiparetic stroke through gait training is the primary goal for stroke rehabilitation (Jette et al., 2005; Latham et al., 2005). Robot-assisted gait training (RAGT) has been proposed as a method for improving the gait of individuals with hemiparetic stroke (Mehrholz et al., 2020), as it can provide intensive, repetitive, task-oriented training for those who are unable to walk independently by partially or fully supporting their weight and movements with a robot control mechanism (Morone et al., 2017). According to systematic reviews, RAGT in individuals with hemiparetic stroke is particularly effective in achieving walking independence (Mehrholz et al., 2020), and its use is recommended in treatment guidelines (Calabrò et al., 2021).

Appropriate assessment of gait characteristics in individuals with hemiparetic stroke is useful for planning treatment goals (Mulroy et al., 2003), monitoring treatment effects (Toro et al., 2003), and predicting the degree of improvement (Kaczmarczyk et al., 2012). Therefore, an important component for maximizing the effectiveness of RAGT is the appropriate assessment of the individual’s gait characteristics during gait training. Observational gait analysis is generally used in clinical practice to analyze gait characteristics (Perry et al., 2010). In contrast, the gait analysis method using a three-dimensional (3D) gait analysis device can acquire objective information, such as temporal and spatial parameters (Baker, 2006). Gait robots have the potential to contribute to quantitatively analyze gait characteristics using mounted sensors; however, no systems have been proposed to analyze gait performance during RAGT.

We developed a new gait training robot with a markerless motion capture system, Welwalk WW-2000 (WW-2000, Toyota Motor Corporation, Aichi, Japan), which enables measurement of various parameters, including time and mechanical assistance load, of an individual’s paralyzed leg. This gait robot system analyzes abnormal hemiparetic gait patterns during RAGT using various sensors (Nakashima et al., 2020). We previously proposed the following index values of nine abnormal gait patterns: hip hiking, circumduction, retropulsion of the hip, excessive hip external rotation, excessive lateral shift of the trunk over the unaffected side, knee extensor thrust, medial whip, posterior trunk tilt, and contralateral vaulting. The index values are highly correlated with those of abnormal gait patterns analyzed using an existing marker-based 3D motion capture system, indicating criterion-related validity (Imoto et al., 2022). In addition to the nine previously reported abnormal gait patterns, the WW-2000 gait analysis system can analyze index values for four more abnormal gait patterns frequently observed in individuals with hemiparetic stroke: anterior trunk tilt (Olney and Richards, 1996), excessive trunk shifts over the affected side (Carr and Shepherd, 1987), excessive knee joint flexion (de Quervain et al., 1996), and swing difficulty (Burpee and Lewek, 2015). There are no reports of objective index values using an existing marker-based 3D motion capture system for these abnormal gait patterns. However, these patterns may lead to unstable walking, decreased walking speed, and a reduced walking endurance in individuals with hemiparetic stroke. Therefore, we needed to examine the validity of the index values analyzed by the WW-2000 gait analysis system using a different research design than that of a previous study (Imoto et al., 2022). The validity of the index values for abnormal gait patterns calculated by the WW-2000 gait analysis system will be comprehensively clarified through the previous study and present study. Consequently, we expect that this gait analysis system will enable the comprehensive assessment for abnormal gait patterns that occur in individuals with hemiparetic stroke during RAGT with quantitative indicators, thereby contributing to the improvement of abnormal gait patterns.

Overall, this study aimed to propose new index values for the four abnormal gait patterns that occur during RAGT, and to clarify the criterion-related validity of the index values of the four new abnormal gait patterns calculated using the WW-2000 gait analysis system: anterior trunk tilt, excessive trunk shifts over the affected side, excessive knee joint flexion, and swing difficulty, in addition to the nine previously reported abnormal gait patterns.

## 2 Methods

### 2.1 Study design and participants

Twelve healthy adults without any musculoskeletal disorders or neurological diseases, the same participants as included in our previous study (Imoto et al., 2022), were enrolled in this criterion-related validity study. The study protocol was approved by the Institutional Review Board (IRB) of Fujita Health University, Japan (IRB approval number: CR23-058) and registered with the Japan Registry for Clinical Trials (jRCTs 042190109) before study enrollment. All the participants provided written informed consent to participate in this study.

### 2.2 Instruments

The WW-2000 supports gait training in individuals with hemiparetic stroke. Individuals wore the robot on their paralyzed lower limbs and walked on a treadmill with robotic assistance. The WW-2000 consists of a knee-ankle-foot type robot, low-floor treadmill, safety suspension system for body weight support, robot suspension system for robot weight support and swing assist, monitor for user, 3D sensors, and control panel. The robot detects the gait cycle using load sensors located on the plantar surface and measures the knee joint angle using a knee angle sensor. On the basis of the data from these sensors, the robot drives and controls the knee joint motor to assist the individual’s knee flexion and extension movements. During the stance phase, it supports knee extension to prevent knee buckling, and this support can be adjusted to 10 levels. During the swing phase, it assists knee flexion and extension to secure clearance. Additionally, it assists in step movements by supporting the weight of the knee-ankle-foot robot, which can be adjusted to six levels.

The components of the markerless motion analysis system in the WW-2000 include a 3D sensor (Xtion2; ASUS Japan Corp., Tokyo, Japan), an inertial sensor (IMU–3 axis inertial sensor AU7684N1; TAMAGAWA SEIKI Co., Ltd., Nagano, Japan), a knee angle sensor, and a load sensor. The 3D sensor was placed 0.6 m above the treadmill surface. The sensor captured color and depth images of the participant’s gait. The two-dimensional joint position coordinates of the participants were estimated from the captured color images using skeleton tracking software (VisionPose®, NEXT-SYSTEM Co., Ltd., Fukuoka, Japan). The 3D joint coordinates were then estimated by adding a depth sensor to the estimated two-dimensional joint position coordinates. An inertial sensor was placed on the thigh of the robot to detect the thigh movement in three directions while walking. A knee angle sensor was installed at the knee joint of the robot to detect the knee joint angle. The system calculated the parameters of abnormal gait patterns common in individuals with hemiparetic stroke using integrated information of the two-dimensional and 3D joint position coordinates and lower limb tilt detected by each sensor.

### 2.3 Experimental tasks

Healthy participants, who were physical therapists with expertise in stroke rehabilitation, simulated four abnormal gait patterns that often manifest in individuals with hemiparetic stroke while wearing the robot on their right leg without a safety suspension system for body weight support. Each participant was instructed to perform 16 gait trials for each of the four grades of severity within each of the four abnormal gait patterns at a distance of 1.2 meters from the 3D sensor. For each gait trial, the participants walked more than 20 strides. The simulated abnormal gait patterns included anterior trunk tilt (Olney and Richards, 1996), excessive trunk shifts over the affected side (Carr and Shepherd, 1987), excessive knee joint flexion (de Quervain et al., 1996), and swing difficulty (Burpee and Lewek, 2015). We designed the setting for the experimental tasks on the basis of a previous study (Imoto et al., 2022). The treadmill speed was set at 0.55 km/h, and the participants were allowed to use the handrail during the assessment. The assistance for the knee joint extension motion in the stance phase and the weight support of the knee-ankle-foot robot in the swing phase were set to the minimum. The flexion and extension angles of the knee joint during the swing phase were maintained at the maximum of 50°. After sharing the definitions and reference movies of abnormal gait patterns, the participants practiced sufficiently simulating the abnormal gait patterns under the instruction by an experienced physical therapist. The participants were asked to simulate four grades of gait patterns, ranging from normal to the most severe, with the same interval of severity between each grade. The measurements were performed after an experienced physical therapist verified that all participants could adequately simulate abnormal gait patterns.

### 2.4 Data acquisition

Gait patterns during the task in healthy adults were recorded using the gait analysis system in WW-2000 and two digital video cameras (NEX-VG30; Sony Marketing Inc., Tokyo, Japan) placed on the side where the knee-ankle-foot robot was worn on the participants’ back. In the gait analysis system in the WW-2000, the 3D joint positions, lower limb tilt, and knee joint angle during the task were recorded using a 3D sensor, inertial sensor, and knee angle sensor, respectively, at a sampling frequency of 30 Hz. The 3D coordinates of the joint positions were estimated from the two-dimensional joint positions obtained using the skeletal tracking software combined with depth information obtained from the 3D sensor. The estimated 3D joint positions were both shoulder, hip, knee, and ankle joints and the midpoints of both shoulder and hip joints. The lower limb tilt and knee joint angle were detected using an inertial sensor located on the thigh and a knee angle sensor located at the knee joint of the robot, respectively. The pitch and roll angles were calculated as the tilt of the lower limb using an algorithm that hybridized the gyrosensor and accelerometer signals built into the inertial sensor (TAMAGAWA SEIKI Co., Ltd, 2022). Load sensors located on the sole of the robot were used to determine the stance phase of the gait cycle. The load sensor was calibrated before participants wore the robot. The knee angle sensor was calibrated while the participants stood with the robot. The 3D and inertial sensors were calibrated with participants in a static standing posture before walking. On the other hand, two digital video cameras recorded the simulated gait patterns at a sampling frequency of 30 Hz.

### 2.5 Data processing and statistical analysis

The abnormal gait pattern index values were calculated for five strides, excluding the first five and last 10 steps of the recorded 20 strides. These values were derived from the definitions provided in Table 1 using the 3D joint positions, tilt of the knee-ankle-foot robot, and knee joint angle of the knee-ankle-foot robot recorded using 3D, inertial, knee angle, and load sensors, respectively.

**Table 1:** Definition of the index values of abnormal gait patterns calculated by the Welwalk WW-2000 gait analysis system.

Abnormal gait pattern	Definition
Anterior trunk tilt	Maximum angle of anterior tilt of the trunk during stance phase on the paralyzed side
Excessive trunk shifts over the affected side	Maximum angle of tilting of the paralyzed thigh in the direction of the paralyzed side during the stance phase of the paralyzed side
Excessive knee joint flexion	Maximum angle of knee flexion during the stance phase on the paralyzed side
Swing difficulty	Mean anteroposterior distance between the hip and ankle joints of the paralyzed side in the terminal swing phase on the paralyzed side

Objective index values indicating the characteristics and severities of these four abnormal gait patterns have not been proposed to date. Therefore, we referenced a previous study (Itoh et al., 2012) and had assessors evaluate the observational gait analysis scores. The specific methods were as follows. The video taken with the digital video camera was edited one step at a time, from the start of the swing phase on the affected side to the start of the next swing phase, using the video software iMovie (Apple Inc., Cupertino, CA, USA). For each participant, a video of five strides was created for each of the four severity grades, resulting in a total of 20 strides per participant. With 12 participants, this amounted to 240 strides, which were then arranged in random order using a random number table. Three physical therapists specializing in gait analysis independently observed and scored each of the 240 strides as normal, mild, moderate, or severe. A study reported that, regarding observational gait analysis, the inter-rater agreement rate for severity assessment was low, while the method was useful as a relative assessment of severity (Tanikawa et al., 2019). Therefore, we designed the procedure for observational gait analysis as follows. Before viewing the videos, the assessors were provided with definitions of each abnormal gait pattern. The median number of years of clinical experience of the assessors was 5 years (range, 5–6 years). For analysis, these scoring results were assigned a numerical value ranging from 1 (normal) to 4 (severe). Videos taken from the right side were used to score the abnormal gait patterns of anterior trunk tilt, excessive knee flexion, and swing difficulty. The video taken from the participant’s back was used to score excessive trunk shifts over the affected side. The assessors were allowed to view the video repeatedly. However, pausing, slow-motion playback, or discussions among the assessors were not permitted. The median values of the gait observation rating were calculated by three assessors who scored the severity of the abnormal gait patterns to examine the relationship with the index values analyzed by the WW-2000 gait analysis system. Cohen weighted kappa coefficients were calculated to assess the inter-rater reliability of the scores. We defined the strength of weighted kappa coefficient as follows: poor agreements, <0.00; slight agreement, 0.00–0.20; fair agreement, 0.21–0.40; moderate agreement, 0.41–0.60; substantial agreement, 0.61–0.80; and almost agreement, 0.81–1.00 (Landis and Koch, 1977).

To confirm the relationship between the severity score based on gait observation and the index values analyzed by the WW-2000 gait analysis system for each abnormal gait pattern, we evaluated the correlations of the index values between the two methods using Spearman rank correlation coefficients for each abnormal gait pattern in each participant. Then, the minimum, median, and maximum values of the correlation coefficients for all the participants were calculated for each gait pattern. We defined the strength of the correlation coefficient as follows: slight correlation, <0.20; low correlation, 0.20–0.39; moderate correlation, 0.40–0.69; high correlation, 0.70–0.89; and very high correlation, >0.90 (Guilford, 1942). Furthermore, the differences in the index values among each severity group for each abnormal gait pattern were compared using the Kruskal–Wallis test, followed by the Steel–Dwass test as a post-hoc analysis. All statistical analyses were performed using EZR (Saitama Medical Center, Jichi Medical University, Saitama, Japan), a graphical user interface for R (The R Foundation for Statistical Computing, Vienna, Austria) (Kanda, 2013).

## 3 Results

Twelve healthy adults without musculoskeletal disorders or neurological diseases participated in this study; of these, six were male. Participants’ mean (standard deviation) age, height, and weight were 27 (3) years, 165 (8) cm, and 56 (7) kg, respectively.

**Figure 1:**
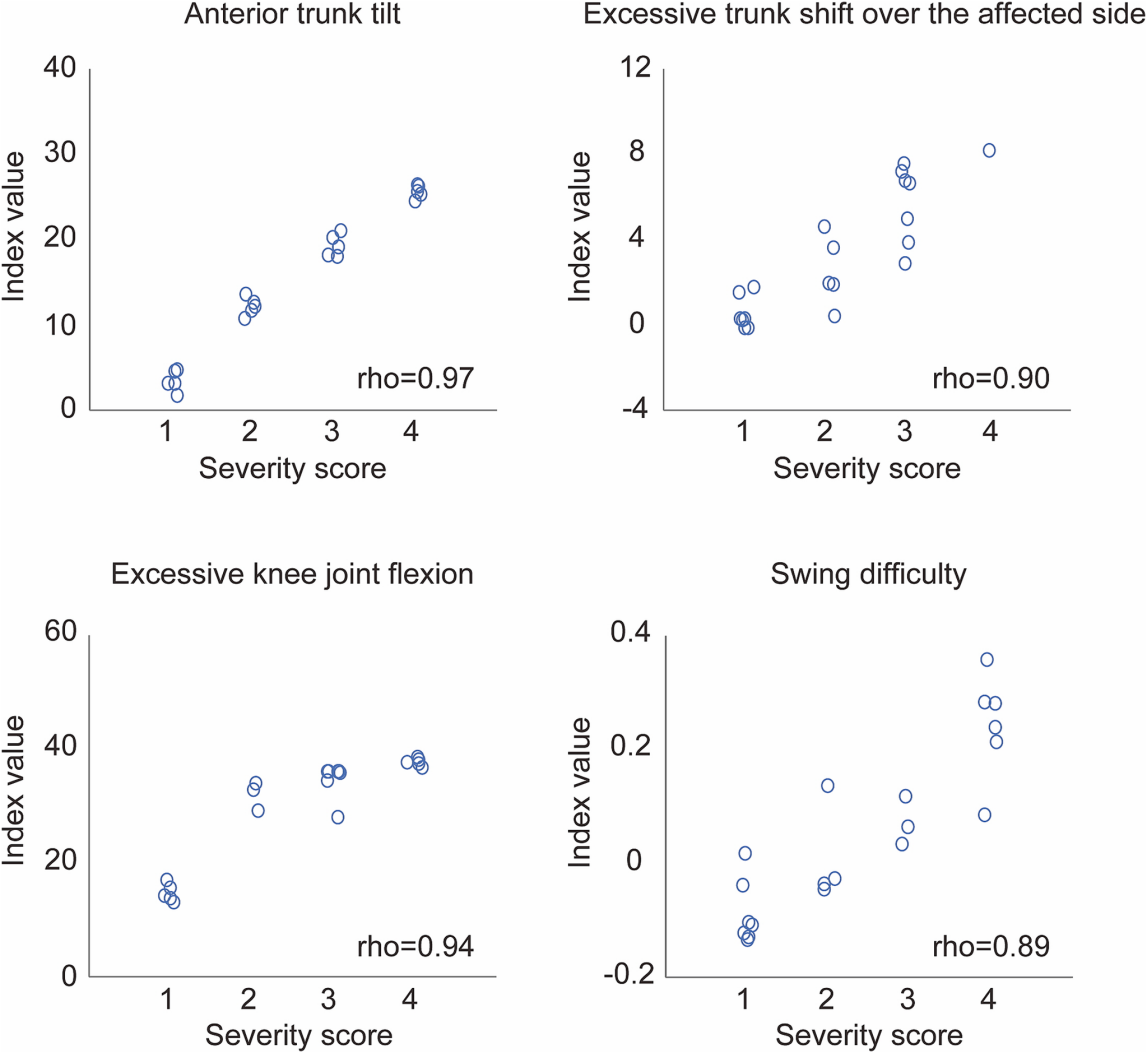
Scatter plots of the index values calculated by the Welwalk WW-2000 gait analysis system and the scoring values obtained by observational gait analysis in a typical case. The severity score on the x-axis shows the median score of the three assessors who scored abnormal gait patterns on a scale from 1 (normal) to 4 (severe).

Scatter plots of the index values analyzed by the WW-2000 gait analysis system and the assessor’s severity scores assessed by observational gait analysis in a typical case are shown in Figure 1. Positive correlations were observed between the index values analyzed by the WW-2000 gait analysis system and the assessor’s severity scores for each abnormal gait pattern. For each abnormal gait pattern, the index values increased according to the assessor’s severity scores (Table 2). The median (minimum to maximum) values of correlation coefficients between the index values calculated by the WW-2000 gait analysis system and the assessor’s severity scores assessed by observational gait analysis for anterior trunk tilt, excessive trunk shifts over the affected side, excessive knee joint flexion, and swing difficulty were 0.892 (0.749–0.969), 0.859 (0.439–0.923), 0.920 (0.738–0.969), and 0.681 (0.391–0.889), respectively. Therefore, these results were judged to have correlations ranging from moderate to very high. The weighted kappa coefficients of the assessor’s severity scores assessed by observational gait analysis for anterior trunk tilt, excessive trunk shifts over the affected side, excessive knee joint flexion, and swing difficulty were 0.913, 0.820, 0.901, and 0.607, respectively. Thus, they were judged to have substantial to almost complete agreement.

**Table 2:** Index values of each abnormal gait pattern simulated by participants at four grades of severity.

Abnormal gait pattern	Grade 1	Grade 2	Grade 3	Grade 4	*p*-value
Anterior trunk tilt	4.018 (−6.104– 13.531)	12.006 (6.380–20.830)	17.474 (11.501–25.942)	22.545 (16.943–29.486)	<0.001
Excessive trunk shifts over the affected side	1.698 (−1.831–4.237)	4.421 (0.382–9.234)	6.243 (2.695–10.217)	9.355 (6.178–12.663)	<0.001
Excessive knee joint flexion	9.908 (6.050–29.115)	26.683 (15.125–34.385)	33.775 (26.605–35.790)	36.873 (35.640–39.610)	<0.001
Swing difficulty	−0.085 (−0.155– −0.005)	−0.029 (−0.134–0.093)	0.030 (−0.103–0.137)	0.132 (−0.087–0.356)	<0.001

Data are presented as median (from minimum to maximum) of all strides. The post-hoc test revealed statistically significant differences among all pairs with *p* < 0.001, except for the pair of grades 3 and 4 in anterior trunk tilt with *p* < 0.01.

## 4 Discussion

This study showed that the correlation between the index values of abnormal hemiparetic gait patterns for anterior trunk tilt, excessive trunk shifts over the affected side, excessive knee joint flexion, and swing difficulty analyzed by the gait analysis system in the WW-2000 and the assessor’s severity scores assessed by observation gait analysis were judged to range from 0.681–0.920, indicating a high criterion-related validity.

The high correlation between our proposed WW-2000 gait analysis system and the assessor’s severity scores from observational gait analysis may be attributed to the configuration of sensors used to analyze abnormal gait patterns. A review article reported that markerless motion capture systems do not have sufficiently accurate positions of the joint center and joint angles for clinical applications because of the effects of clothing and posture estimation algorithms (Wade et al., 2022). Another review paper also proposed the creation of a multimodal gait analysis system with inertial sensor units to avoid errors caused by inertial sensor units, such as data drift caused by magnetic fields (Gu et al., 2023). For example, Vargas-Valencia et al. has proposed a motion capture system to measure knee angle combined IMU sensors with polymer optical fiber curvature sensor (Vargas-Valencia et al., 2021). These studies suggested that gait analysis systems that use a single sensor have limitations in capturing an individual’s gait characteristics. We assume that the gait analysis system proposed here was more accurate as multiple-sensor information was integrated.

The clinical significance of this research is that we have proposed a system to analyze the severity of abnormal gait patterns during RAGT in real time based on objective indicators. This is a markerless motion capture gait analysis system that eliminates the need for physical therapists to spend time on gait analysis allowing them to simultaneously conduct gait training and analyses in individuals. Using this system, RAGT tailored to the individual can be conducted on the basis of indicators of the analyzed abnormal gait patterns. This system has the potential to contribute to immediate clinical decision-making based on objective indicators and to provide individualized RAGT. Future research is expected to clarify the relationship between abnormal gait patterns during training and training settings, e.g., robotic assistance, leading to individual-specific gait training.

This study has some limitations. First, it was difficult to recruit a large number of individuals with hemiparetic stroke who had various severities of abnormal gait patterns. This difficulty arose because individuals with severe gait disturbance typically require assistance, whereas those who do not require assistance rarely exhibit severe abnormal gait patterns. Consequently, simulated gait patterns in healthy adults were used in the experiment. Therefore, we ensured that the participants were thoroughly instructed by therapists with expertise in stroke rehabilitation. However, the biomechanical changes caused by functional disorders, such as weakness and spasticity, that occur in individuals with hemiparetic stroke cannot be completely simulated, which may affect the index values. Future research should take steps to examine the validity of the system among individuals with hemiparetic stroke. Second, while there is a gold standard for measuring trunk and joint movements (Rosa et al., 2015; Van Criekinge et al., 2017; Wang et al., 2017), there is no objective index to provide a gold standard for the abnormal gait patterns targeted in this study, particularly for overall movements like those examined here. Thus, scoring results from observation gait analysis were used in the present study. We performed procedures to improve the agreement rate among assessors, referring to the findings of a previous study (Tanikawa et al., 2019). Additionally, we checked the agreement rate among assessors’ scores. Consequently, the agreement of the assessors’ scoring ranged from substantial to almost complete agreement. Therefore, we believe that the present study design was not a critical issue.

## 5 Conclusion

This study showed that the WW-2000 gait analysis system analyzed four unconfirmed abnormal gait patterns–anterior trunk tilt, excessive trunk shifts over the affected side, excessive knee joint flexion, and swing difficulty–during RAGT with high criterion-related validity. Assessing abnormal gait patterns is important as improving these patterns contributes to gait rehabilitation for individuals with hemiparetic stroke.

## Data Availability

The raw data supporting the conclusions of this article will be made available by the authors, without undue reservation.

## References

[ref9001] Baker R. (2006). Gait analysis methods in rehabilitation. J. Neuroeng. Rehabil. 3:4. doi: 10.1186/1743-0003-3-4 16512912 PMC1421413

[ref1] Burpee J. L. Lewek M. D. (2015). Biomechanical gait characteristics of naturally occurring unsuccessful foot clearance during swing in individuals with chronic stroke. Clin. Biomech 30, 1102–1107. doi: 10.1016/j.clinbiomech.2015.08.018, PMID: 26371855

[ref2] Calabrò R. S. Sorrentino G. Cassio A. Mazzoli D. Andrenelli E. Bizzarini E.. (2021). Robotic-assisted gait rehabilitation following stroke: a systematic review of current guidelines and practical clinical recommendations. Eur. J. Phys. Rehabil. Med. 57, 460–471. doi: 10.23736/s1973-9087.21.06887-8, PMID: 33947828

[ref3] Carr J. H. Shepherd R. B. (1987). A motor relearning programme for stroke. Rockville, MD: Aspen Publs.

[ref4] de Quervain I. A. K. Simon S. R. Leurgans S. Pease W. S. McAllister D. (1996). Gait pattern in the early recovery period after stroke. J. Bone Joint Surg. 78, 1506–1514. doi: 10.2106/00004623-199610000-00008, PMID: 8876578

[ref5] Gu C. Lin W. He X. Zhang L. Zhang M. (2023). IMU-based motion capture system for rehabilitation applications: a systematic review. BIROB 3:100097. doi: 10.1016/j.birob.2023.100097

[ref6] Guilford J. P. (1942). Fundamental statistics in psychology and education. New York, NY: McGraw-Hill.

[ref8] Imoto D. Hirano S. Mukaino M. Saitoh E. Otaka Y. (2022). A novel gait analysis system for detecting abnormal hemiparetic gait patterns during robot-assisted gait training: a criterion validity study among healthy adults. Front. Neurorobot. 16:1047376. doi: 10.3389/fnbot.2022.1047376, PMID: 36531918 PMC9751383

[ref9] Itoh N. Kagaya H. Saitoh E. Ohtsuka K. Yamada J. Tanikawa H.. (2012). Quantitative assessment of circumduction, hip hiking, and forefoot contact gait using Lissajous figures. Jpn. J. Comprehensive Rehab. Sci. 3, 78–84. doi: 10.11336/jjcrs.3.78

[ref10] Jette D. U. Latham N. K. Smout R. J. Gassaway J. Slavin M. D. Horn S. D. (2005). Physical therapy interventions for patients with stroke in inpatient rehabilitation facilities. Phys. Ther. 85, 238–248. doi: 10.1093/ptj/85.3.238 15733048

[ref11] Kaczmarczyk K. Wit A. Krawczyk M. Zaborski J. Gajewski J. (2012). Associations between gait patterns, brain lesion factors and functional recovery in stroke patients. Gait Posture 35, 214–217. doi: 10.1016/j.gaitpost.2011.09.009, PMID: 21937234

[ref12] Kanda Y. (2013). Investigation of the freely available easy-to-use software ‘EZR’ for medical statistics. Bone Marrow Transplant. 48, 452–458. doi: 10.1038/bmt.2012.244, PMID: 23208313 PMC3590441

[ref13] Landis J. R. Koch G. G. (1977). The measurement of observer agreement for categorical data. Biometrics 33, 159–174. doi: 10.2307/2529310 843571

[ref14] Latham N. K. Jette D. U. Slavin M. Richards L. G. Procino A. Smout R. J.. (2005). Physical therapy during stroke rehabilitation for people with different walking abilities. Arch. Phys. Med. Rehabil. 86, 41–S50. doi: 10.1016/j.apmr.2005.08.128, PMID: 16373139

[ref15] Mehrholz J. Thomas S. Kugler J. Pohl M. Elsner B. (2020). Electromechanical-assisted training for walking after stroke. Cochrane Database Syst. Rev. 2020:CD006185. doi: 10.1002/14651858.CD006185.pub5, PMID: 33091160 PMC8189995

[ref16] Morone G. Paolucci S. Cherubini A. De Angelis D. Venturiero V. Coiro P.. (2017). Robot-assisted gait training for stroke patients: current state of the art and perspectives of robotics. Neuropsychiatr. Dis. Treat. 13, 1303–1311. doi: 10.2147/NDT.S114102, PMID: 28553117 PMC5440028

[ref17] Mulroy S. Gronley J. Weiss W. Newsam C. Perry J. (2003). Use of cluster analysis for gait pattern classification of patients in the early and late recovery phases following stroke. Gait Posture 18, 114–125. doi: 10.1016/s0966-6362(02)00165-0, PMID: 12855307

[ref9002] Nakashima I. Imoto D. Hirano S. Mukaino M. Imaida M. Saitoh E.. (2020). Development of an abnormal gait analysis system in gait exercise assist robot “Welwalk” for hemiplegic stroke patients, in Proceedings of the 8th IEEE RAS/EMBS international conference for biomedical robotics and biomechatronics (BioRob), (Piscataway, NJ: IEEE), 1030–1035. doi: 10.1109/BioRob49111.2020.9224323

[ref18] Olney S. J. Richards C. (1996). Hemiparetic gait following stroke. Part I 4, 136–148. doi: 10.1016/0966-6362(96)01063-6

[ref7001] Perry J. Burnfield J. M. (2010). Gait analysis: normal and pathological function (version second edition). Thorofare, NJ: SLACK.

[ref19] Rosa M. C. N. Marques A. Demain S. Metcalf C. D. (2015). Knee posture during gait and global functioning post-stroke: a theoretical ICF framework using current measures in stroke rehabilitation. Disabil. Rehabil. 37, 904–913. doi: 10.3109/09638288.2014.948132, PMID: 25095902

[ref20] TAMAGAWA SEIKI Co., Ltd. (2022). *MEMS IMU*. Available online at: https://mems.tamagawa-seiki.com/en/product/memsimu.html#p03 (Accessed July 1, 2024).

[ref23] Tanikawa H. Ohtsuka K. Yamada J. Mukaino M. Matsuda F. Kagaya H.. (2019). Influence of clinical experience and instruction on typical cases on the inter-rater reliability of observational gait analysis. Jpn. J. Comprehensive Rehab. Sci. 10, 14–20. doi: 10.11336/jjcrs.10.14

[ref24] Toro B. Nester C. J. Farren P. C. (2003). The status of gait assessment among physiotherapists in the United Kingdom. Arch. Phys. Med. Rehabil. 84, 1878–1884. doi: 10.1016/s0003-9993(03)00482-9, PMID: 14669198

[ref25] Van Criekinge T. Saeys W. Hallemans A. Velghe S. Viskens P. J. Vereeck L.. (2017). Trunk biomechanics during hemiplegic gait after stroke: a systematic review. Gait Posture 54, 133–143. doi: 10.1016/j.gaitpost.2017.03.004, PMID: 28288334

[ref9003] Vargas-Valencia L. S. Schneider F. B. Leal-Junior A. G. Caicedo-Rodriguez P. Sierra-Arevalo W. A. Rodriguez-Cheu L. E.. (2021). Sleeve for knee angle monitoring: an IMU-POF sensor fusion system. IEEE J Biomed Health Inform. 25, 465–474. doi: 10.1109/jbhi.2020.2988360 32324580

[ref26] Wade L. Needham L. McGuigan P. Bilzon J. (2022). Applications and limitations of current markerless motion capture methods for clinical gait biomechanics. PeerJ 10:e12995. doi: 10.7717/peerj.12995, PMID: 35237469 PMC8884063

[ref27] Wang W. Li K. Yue S. Yin C. Wei N. (2017). Associations between lower-limb muscle activation and knee flexion in post-stroke individuals: a study on the stance-to-swing phases of gait. PLoS One 12:e0183865. doi: 10.1371/journal.pone.0183865, PMID: 28886079 PMC5590852

